# Comparison of proteome response to saline and zinc stress in lettuce

**DOI:** 10.3389/fpls.2015.00240

**Published:** 2015-04-16

**Authors:** Luigi Lucini, Letizia Bernardo

**Affiliations:** Institute of Environmental and Agricultural Chemistry, Università Cattolica del Sacro CuorePiacenza, Italy

**Keywords:** osmotic stress, oxidative stress, shotgun MS, photosystem I, phytohormone

## Abstract

Zinc salts occurring in soils can exert an osmotic stress toward plants. However, being zinc a heavy metal, some more specific effects on plant metabolisms can be forecast. In this work, lettuce has been used as a model to investigate salt and zinc stresses at proteome level through a shotgun tandem MS proteomic approach. The effect of zinc stress in lettuce, in comparison with NaCl stress, was evaluated to dissect between osmotic/oxidative stress related effects, from those changes specifically related to zinc. The analysis of proteins exhibiting a fold change of 3 as minimum (on log 2 normalized abundances), revealed the involvement of photosynthesis (via stimulation of chlorophyll synthesis and enhanced role of photosystem I) as well as stimulation of photophosphorylation. Increased glycolytic supply of energy substrates and ammonium assimilation [through formation of glutamine synthetase (GS)] were also induced by zinc in soil. Similarly, protein metabolism (at both transcriptional and ribosomal level), heat shock proteins, and proteolysis were affected. According to their biosynthetic enzymes, hormones appear to be altered by both the treatment and the time point considered: ethylene biosynthesis was enhanced, while production of abscisic acid was up-regulated at the earlier time point to decrease markedly and gibberellins were decreased at the later one. Besides aquaporin PIP2 synthesis, other osmotic/oxidative stress related compounds were enhanced under zinc stress, i.e., proline, hydroxycinnamic acids, ascorbate, sesquiterpene lactones, and terpenoids biosynthesis. Although the proteins involved in the response to zinc stress and to salinity were substantially the same, their abundance changed between the two treatments. Lettuce response to zinc was more prominent at the first sampling point, yet showing a faster adaptation than under NaCl stress. Indeed, lettuce plants showed an adaptation after 30 days of stress, in a more pronounced way in the case of zinc.

## Introduction

Salt stress and NaCl in particular, is probably the most common abiotic stress affecting crop production and reducing yields. In response to salinity, plants evolved specific mechanisms to sense and contrast this stress at both metabolite and protein level. The proteins identified in different studies are involved in the major plant metabolic processes, such as photosynthesis, energy metabolism, ROS scavenging, and ion homeostasis, protein synthesis, nitrogen assimilation as well as the secondary oxidative stress (Parida and Das, 2005; Zhang et al., 2012). High levels of NaCl can be toxic for plants and cause stunted growth together with reduction in water potential (Borgognone et al., 2013).

Conversely, Zn^2+^ is an essential micronutrient for plants, and it is therefore essential for plant growth, development, and many metabolic processes being one of the major cofactors, together with iron and manganese, in numerous enzymes (Andreini et al., 2008). More than 1200 proteins are predicted to contain, bind, or transport Zn^2+^ (Hänsch and Mendel, 2009). Furthermore, the zinc-inorganic phosphate (Pi) relationship has been observed in numerous plant species such as tomato, cotton, barley, and *Arabidopsis* (Bouain et al., 2014). Besides being a micronutrient, zinc is also a heavy metal and can have detrimental effects on many vital processes in plant cells. Geological and/or anthropogenic activities can result in zinc concentration in soil above toxic levels for crops, leading to chlorosis, biomass reduction, and necrotic lesions on leaves. Nowadays, few studies have investigated the effects of zinc stress to plants by a proteomic approach. Zinc toxicity effects have been mainly investigated in roots of model plants by means of gel-based proteomic approaches (Gutierrez-Carbonell et al., 2013; Romeo et al., 2014). In *Arabidopsis thaliana* plants exposed to zinc at sub-toxic levels, Barkla et al. (2014) found that the enzymes involved in one carbon metabolism and protein synthesis were involved in acclimation to heavy metal stress. At higher concentration, this species showed alteration of proteins related to oxidative stress, proteasome, and energy metabolism (Fukao et al., 2009). Even if a few studies dealing with hyper-accumulating plants have been reported (Barkla et al., 2014; Lefèvre et al., 2014), the knowledge about zinc toxicity in crops is still limited. Most of the work in agricultural species work has been done in sugar beet, demonstrating the imbalance of photosystems, oxidative stress, alteration of carboxylates trafficking, and low photosynthesis rates as a consequence of stomatal and mesophyll conductance to CO_2_ (Sagardoy et al., 2009, 2010, 2011).

Lettuce (*Lactuca sativa* L.) is one of the most common fresh-cut vegetables in the Mediterranean diet, containing health-promoting phytochemicals such as phenolic compounds, vitamin C, and carotenoids (Garrido et al., 2014). This species has been proposed as a good model to study the zinc influence on plant growth, showing differential biomass and photosynthesis rate (Bouain et al., 2014). The zinc toxicity in lettuce has been related to carboxylate metabolism (Barrameda-Medina et al., 2014). However, lettuce is also moderately sensitive to salt stress, and exhibits reduced growth under saline conditions. Kim et al. (2008) have investigated the secondary metabolite profile of lettuce under high NaCl concentration, observing that biomass was reduced above 100 mM NaCl soil concentration for 15 days, in comparison to untreated plants.

Although plant response to salinity has been widely investigated in many species at protein level (reviewed by Zhang et al., 2012; Hossain and Komatsu, 2013), very little and fragmentary information is available regarding the response to zinc exposure. In this work, lettuce has been used as a model to investigate and compare salt and zinc stresses, aimed to understand the differences at proteome level and hence the modulation of metabolic pathways. In the study, a gel-free bottom-up proteomic approach was chosen to provide understanding of the molecular mechanisms underlying plant adaptation to zinc-contaminated soils, in comparison to salinity stress. Despite zinc salts in soil can represent a plant stressor related to changes in osmolality and water availability, as well as salinity does, this heavy metal might induce some additional and more specific responses. The assessment of the responses in common between the two stresses, together with the disjoint ones, can provide useful insights on the plant response to either zinc or salt contaminated soil, thus supporting crop production, and promoting a more efficient land use.

## Materials and Methods

### Plant Material and Mineral Analysis

Young plants of lettuce (*L. sativa* L. cultivar longifolia), about 15 cm in length, were purchased at a local nursery and transplanted into large pots (nine plants per pot at 6 cm distance, in a 36 L pot) filled with a commercial topsoil. Overall, six pots were prepared: two serving as control, two for NaCl, and two for zinc treatments, respectively. Plants were grown indoor, under natural light and at room temperature.

After 1 week of acclimation, two pots were irrigated with demineralized water (control), two with a 100 mM NaCl solution, and two with a 100 mM ZnSO_4_ solution, with the same volume twice a week. After both 15 and 30 days of treatment, a pot per treatment was withdrawn and three plant leaves per pot collected and pooled as biological replications. Therefore, three replicate samples of leaves (each of them taken from three plants) were harvested per treatment, at both 15 and 30 days.

Leaf samples were frozen in liquid N_2_ and ground into a fine powder using a mortar and pestle. A portion (100 mg) of each sample was mineralized in 0.5 mL of hydrogen peroxide and 2 mL of nitric acid, under heating for 6 h, diluted in 5% nitric acid, and then analyzed by inductively coupled plasma atomic emission spectroscopy. A multi-element source was used for calibration purposes and a certified reference material was analyzed before samples; each sample was read in duplicate.

### Protein Extraction

Tissue powder (125 mg) was suspended in 0.8 mL ice-cold SDS-phenol buffer (0.1 M Tris-HCl pH 8.0, 30% sucrose, 2% SDS, 2% DTT) and equal volume of Tris-buffered phenol pH 8.0, shaken and centrifuged at 15000 *g* for 5 min at 4°C. The phenolic phase was collected and proteins were precipitated with 5 volumes of 0.1 M ammonium acetate in methanol, overnight at -20°C. Samples were centrifuged for 15 min to 10000 *g* at 4°C and then pellets were washed three times with 80% acetone. Finally, the dry pellets were resuspended in a buffer containing 7 M urea and 2 M thiourea. The protein concentration was measured with the Bio-Rad protein assay kit using bovine γ-globulin as standard, following to the manufacturer’s instructions. Protein extracts were stored at -20°C until use. Fifty micrograms of proteins were then reduced with dithiothreitol, alkylated with iodoacetamide, and digested with Trypsin (Promega, Madison, WI, USA) at 37°C overnight.

### Tandem MS Analysis

Tryptic peptides were analyzed by a shotgun MS/MS approach using a hybrid quadrupole-time-of-flight (Q-TOF) mass spectrometer. With this purpose, an Agilent 6550 IFunnel Q-TOF mass spectrometer, with a nano LC Chip Cube source (Agilent Technologies, Santa Clara, CA, USA), was used. The chip consisted of a 40-nL enrichment column (Zorbax 300SB-C18, 5 μm pore size) and a 150 mm separation column (Zorbax 300SB-C18, 5 μm pore size) coupled to an Agilent Technologies 1200 series nano/capillary LC system and controlled by the MassHunter Workstation Acquisition (version B.04).

Peptides were loaded onto the trapping column at 2.6 μL min^-1^ in 2% (v/v) acetonitrile and 0.1% (v/v) formic acid. After enrichment, the chip was switched to separation mode and peptides were backflush eluted into the analytical column, during a 150 min acetonitrile gradient (from 3 to 70% v/v) in 0.1% (v/v) formic acid at 0.3 μl min^-1^. The mass spectrometer was used in positive ion mode and MS scans were acquired over a range from 300 to 1700 mass-to-charge ratio at 4 spectra s^-1^. Precursor ions were selected for auto-MS/MS at an absolute threshold of 1000 and a relative threshold of 0.01%, with a maximum of 20 precursors per cycle and active exclusion set at 2 spectra (with release after 0.2 min). Analysis of MS/MS spectra for peptides identification was performed by protein database searching with Spectrum Mill MS Proteomics Workbench (Rev B.04; Agilent Technologies). Auto MS/MS spectra were extracted from raw data accepting a minimum sequence length of three amino acids and merging scans with the same precursor within a mass window of ±0.4 mass-to-charge ratio in a time frame of ±30 s. Search parameters were Scored Peak Intensity (SPI) ≥50%, precursor mass tolerance of ±10 ppm and product ions mass tolerance of ±20 ppm. Carbamidomethylation of cysteine was set as fixed modification and trypsin was selected as enzyme for digestion, accepting two missed cleavages per peptide. The search was conducted against the proteome of *L. sativa* (UniProt, downloaded October 2014); the database was concatenated with the reverse one. Auto thresholds were used for peptide identification in Spectrum Mill to achieve a target 1% false discovery rate. A label-free quantitation, using the protein summed peptide abundance, was carried out after identification.

The results were directly exported to Mass Profiler Professional B.04 (Agilent Technologies) for statistics and pathway analysis. Protein intensities were log 2 normalized and baselined versus the control, and then ANOVA and fold-change analysis were done. Those proteins showing a normalized fold-change above 3 were considered and finally linked to *A. thaliana* metabolic pathways (source: WikiPathways) in the Pathway Architect add-in of Mass Profiler Professional, using their Uniprot ID annotation.

Differential proteins identified from Swiss-Prot were searched against AgriGO analysis tool to assign functional ontology information. The proteins with associated GOs were then loaded in REVIGO to visualize proteins GOs frequencies into functional processes against all *A. thaliana* GOs.

## Results

**FIGURE 1:**
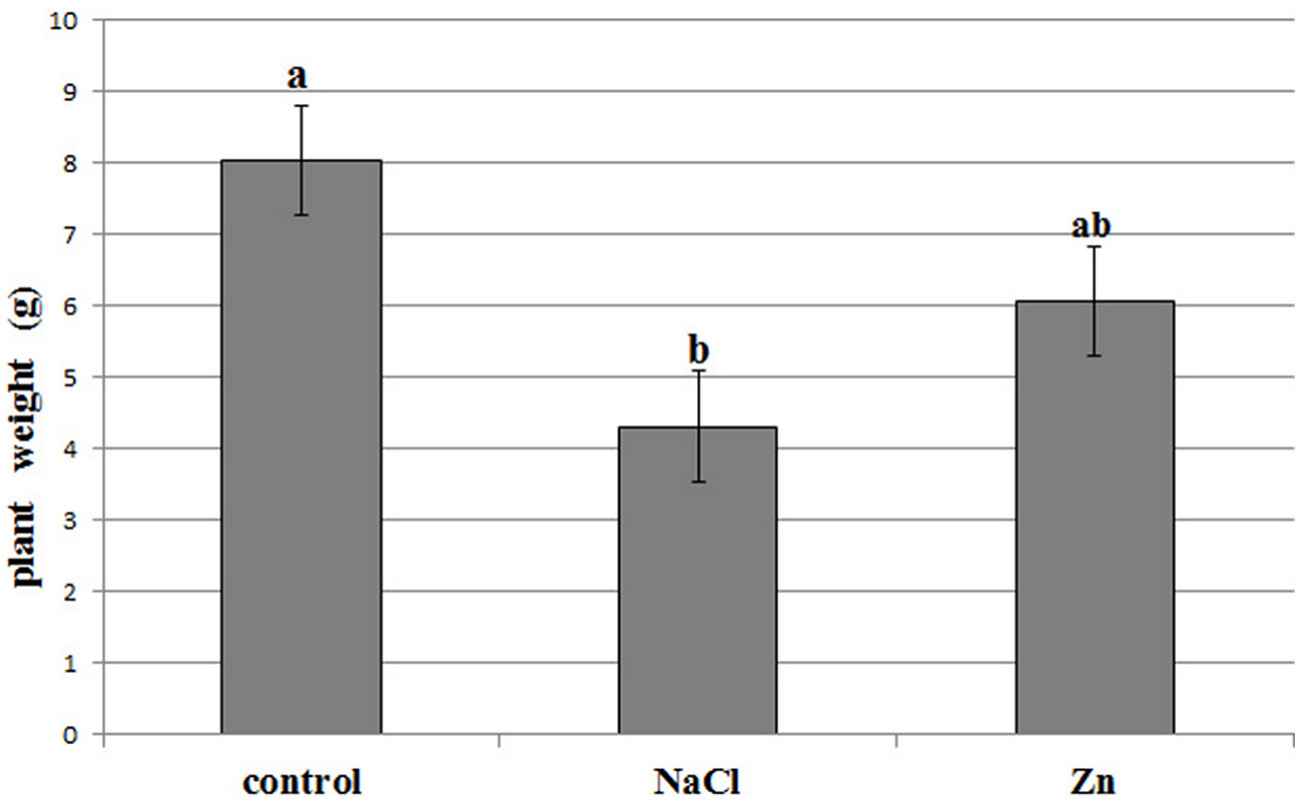
**Biomass production by the control, zinc stressed, and NaCl stressed treatments at harvest, after 30 days from stress application**. Lowercase letters indicate statistically significant differences (ANOVA, α = 0.05).

The different treatments affected the production of biomass, as plant mean weight was significantly reduced under saline conditions (**Figure 1**), whereas the detrimental effect of zinc on yield was less evident (ANOVA at alpha = 0.05, Student–Newman–Keuls *post hoc* test). However, besides the above mentioned reduction in yield, no symptoms of toxicity could be evidenced at phenotype level. Metals analysis in plant leaves, investigated through inductively coupled plasma atomic emission spectroscopy, revealed that zinc and sodium concentration increased as a consequence of the corresponding treatment. Zinc was accumulated in leaves, raising up to 468 mg/kg at fresh weight (three times and 12 times the level in control, after 15 and 30 days of treatment, respectively), while sodium levels reached 3.5% of leaf fresh weight (four times and 27 times the level in control, after 15 and 30 days of treatment, respectively). Therefore, both Zn^2+^ and Na^+^ were actually translocated to leaves, supporting metal-related variations at proteome level.

**FIGURE 2:**
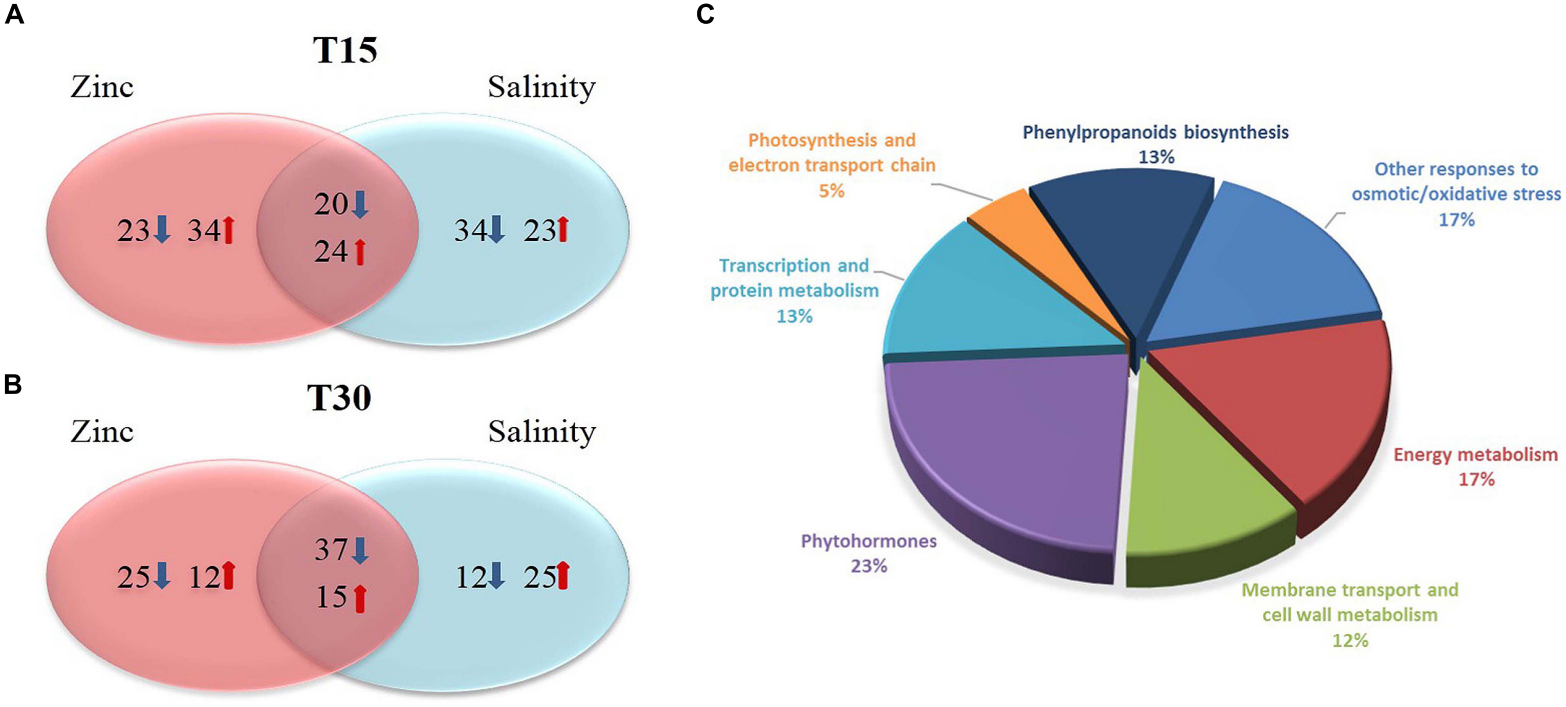
**Venn diagrams of the proteins resulting up- or down-accumulated after 15 and 30 days (A, upper and B, lower pane for earlier and later time point, respectively), together with the classification into functional ontology (C)**. Fold change analysis was carried out on log 2 normalized abundances, using a cut off = 3. The arrows indicate the number of proteins increasing or decreasing their abundance (in red and blue, respectively).

Changes at proteome level were investigated through a shotgun MS approach, with label-free quantitation. Only those proteins passing the validation step done using the Spectrum Mill search engine (auto-thresholds, FDR 1%), have been considered. Single peptide identification was conditional to the use of unique peptides, then the following filtering in Mass Profiler Professional let to identify and report those proteins being detected in two out of three replications, as minimum. Overall, 124 and 122 identified proteins were validated at the first (15 days) and second (30 days) sampling point, respectively. This dataset was then subjected to statistics and fold-change analysis. **Figure 2** summarizes the proteins detected in each treatment together with their functional ontology, while a detailed list of all identified proteins with their abundances and score is given as Supplementary Material.

Several studies are present in literature to uncover the plant response to salinity at molecular and physiological level. Salinity leads to a cellular osmotic adjustment to hinder the toxic effects of Na^+^ and alters the salt-responsive proteins (involved in photosynthesis, energy metabolism, ROS scavenging, and ion homeostasis; Zhang et al., 2012). The increase in superoxide dismutase (SOD) and phenylalanine ammonia-lyase (PAL) played a key role to cope with the oxidative stress related to salinity. Several proteins involved in protein synthesis, turnover, and degradation were up accumulated in our experiments [i.e., ribosomal proteins, heat shock proteins, maturase K (matK)] together with proteins involved in hormonal signaling and in ethylene metabolism, such as the cysteine-rich RLK protein, 1-aminocyclopropane-1-carboxylic acid (ACC) synthase 1 (ACS1), ACC oxidase (ACO) and the transcription factor MYC2. Although the differential proteins identified from the salt-stress treatment (**Table 1**) were in common with those from zinc-stress, differences could be noted in abundance profiles and trend across time points. Our results from the NaCl trial are in agreement with previously published studies (Golldack et al., 2014). No novel findings were pointed out in comparison with previous works; therefore, our results are mainly focused on zinc-related changes at proteome level, while less attention has been given to salinity. Results are reported at two time points, 15 and 30 days after stress application, in order to point out an eventual adaptation of lettuce plant to high zinc concentrations in soil.

**Table 1:** Proteins resulted down-accumulated and up-accumulated in lettuce leaf after 15 and 30 days under zinc stress conditions, considering a fold change above three on log 2 normalized intensities.

Protein name	Swiss-Prot ID	Log fold-change (normalized)	
		15 days	30 days
**Photosynthesis and electron transport chain**
37 kDa chloroplast inner envelope membrane protein (Fragment)	Q9SEC0	-0.13317^a,b^	
Far-red impaired response protein-like protein (Fragment)	Q6Y1C2	8.400507	-0.15199^b^
MPBQ/MSBQ transferase	D2D1G3		4.488389^b^
NAD(P)H-quinone oxidoreductase subunit 5, chloroplastic	Q32539	-5.50839^b^	
NAD(P)H-quinone oxidoreductase subunit I, chloroplastic	Q332S3	4.688174	
NAD(P)H-quinone oxidoreductase subunit J, chloroplastic	Q332X5	3.071047^a^	0.786051^a^
NAD(P)H-quinone oxidoreductase subunit K, chloroplastic	Q332X4	-0.13317	
Photosystem I assembly protein Ycf3	Q1KXM2		3.454656
Photosystem I P700 chlorophyll *a* apoprotein A1	Q332X8	0.377403^a,b^	
**Energy metabolism**
Cytosolic fructose-1,6-bisphosphate (Fragment)	Q9SEC1		4.497181^a^
Sucrose:sucrose 1-fructosyltransferase	A9YTS8		-0.37763
Triosephosphate isomerase, cytosolic (Fragment)	P48493	0.652735^b^	-1.429^b^
**Transcription and protein metabolism**
30S ribosomal protein S11, chloroplastic	Q332U4		4.139288
30S ribosomal protein S15, chloroplastic	Q332S0	-0.13317	
30S ribosomal protein S16, chloroplastic	Q332Z6	4.159053	
30S ribosomal protein S19, chloroplastic	Q332T6	-5.81259	
30S ribosomal protein S3, chloroplastic	Q332T8	-0.13317	-0.37763
30S ribosomal protein S3, chloroplastic	Q332T8		-0.37763
30S ribosomal protein S4, chloroplastic	Q332X6	-0.13317	
50S ribosomal protein L14, chloroplastic	Q332U0	-5.12I868^a,b^	
50S ribosomal protein L20, chloroplastic	Q332V5	4.634185	4.494172
50S ribosomal protein L33, chloroplastic	Q332V7	6.178312	
Cystatin (Fragment)	U5HV08	8.643057^a^	
Cysteine protease	E9NVM7	4.293656	
Deoxyhypusine synthase	Q643S4	-0.13317	
DNA-directed RNA polymerase subunit alpha	Q332U5	5.632396	
DNA-directed RNA polymerase subunit beta	Q56P12		-6.22537
Fructokinase-like protein 1	D9IWP4		-0.37763^a,b^
Heat shock protein 70-like protein (Fragment)	Q08II7	4.398442	
Heat shock protein 90	J7KE88	7.922094	-0.92689^a^
Maturase K (matK)	D1LZC1	-0.13317	
Protection of telomeres 1 protein	B7T1J4	1.122434	
Putative ankyrin-like protein (AKR)	A1KXE3	-0.13317^a^	
Putative ribosomal protein L31	A1KXE2	4.333077^a^	-0.37763^b^
Ribosomal protein S10 (Fragment)	Q9FUT9		-0.37763
ZF-HD homeobox protein-like protein (Fragment)	Q6Y1B4	-0.13317	
**Phytohormones**
Nine-*cis*-epoxycarotenoid dioxygenase 1 (NCED1)	Q2PHG2		-0.37763
NCED2	Q2PHG1		-0.6249^b^
NCED3	Q2PHG0	-0.13317	-0.37763
ABA 8-oxidase	B1B1U2	4.783829^b^	
ACC oxidase 1 (ACO1)	B9A0T8		3.793779^b^
Carotenoid cleavage dioxygenase 1 (CCDs)	Q2PHF8	4.179041^a^	-0.37763
CCDs 2	Q2PHF7	-0.13317	
DELLA 2	B5MEX9		7.979673^a^
Ent-kaurene oxidase 1 (KO)	B5MEX5		-0.37763^b^
GA Insensitive Dwarf1 (GID1) A	I2FGB7	-0.13317	
GIGANTEA (GI; Fragment)	F5AAK6		-0.37763
MYC2 (Fragment)	F5ABE6	-0.13317^a^	-0.37763^b^
NCED 4	R4UQH3	4.181489	
Putative ACC synthase 1	Q84QH6	-0.13317^a^	
Putative ACC synthase 2	Q84QH5	5.250052	5.168442
Putative ethylene receptor (ETR1)	Q5K6M7	4.862879	
**Membrane transport and cell wall metabolism**
Pectin acetylesterase (PAE)	A1KXE1	4.286351	
Putative cellulose synthase (CesA; Fragment)	A8QV02		-0.37763
Putative MDR-like P-glycoprotein	A1KXD8	9.416772^a^	
Putative membrane protein ycf1	Q1KXH4	0.087746^b^	-0.37763^a^
Putative PIP2 aquaporin (Fragment)	Q2PBP2		-0.37763
**Phenylpropanoids biosynthesis**
Flavanone-3-beta-hydroxylase (F3H; Fragment)	D7UQM2	6.423115	6.321802
Phenylalanine ammonia-lyase (PAL)	Q8W2E4	-4.36404^b^	
**Response to osmotic/oxidative stress**
Alcohol dehydrogenase (ADH) class III	J7GHV7	3.350566	
ADHs homologous	Q40249	-2.43125^a^	
Costunolide synthase	F8S1I0	4.779782	
Delta 1-pyrroline-5-carboxylate synthetase (P5CS; Fragment)	Q5K3Q5	4.709259	
Germacrene A synthase (GAS) LTC1	Q8S3A6	5.004176	
Isopentenyl pyrophosphate:dimethyllallyl pyrophosphate isomerase (IDI; Fragment)	Q9M6K6		4.79889^b^
L-galactono-1,4-lactone dehydrogenase (L-GalLDH)	W6N8V9		5.031978
NBS-LRR resistance-like protein 4E (Fragment)	B9UNN7		-0.37763^a^
NBS-LRR resistance-like protein RGC1F (Fragment)	B8XDI2		-0.37763
NBS-LRR resistance-like protein RGC8A (Fragment)	B8XDI7	4.433209	
Non-specific lipid-transfer protein	A1E2H5		-0.37763
Resistance protein candidate (Fragment)	Q6YFG5	-0.13317	
Resistance protein candidate RGC2 (Fragment)	Q6Y141	4.220169^a^	
Resistance protein candidate RGC2C (Fragment)	Q9ZSD0	8.49143^a^	
Resistance protein candidate RGC2S	Q9ZSC9	-0.13317	
Resistance protein RGC2	Q6Y140	4.641274	-5.35865^a^
Superoxide dismutase (SOD; Fragment)	Q93YG5	-4.3103	
Tocopherol cyclase (TC)	D6C5X6	-5.27813	-0.37763
**Others**
Chloroplast At1g16080 protein (Fragment)	Q1HRX8	9.766279^a,b^	
Putative uncharacterized protein (Fragment)	C7F9L1		-0.37763
Uncharacterized protein	R4UMR1	4.153258^a^	

*Protein fold changes are listed in the table. ^a^Proteins identified that are positive to PLS-DA statistical analysis. ^b^Proteins identified that define naïve Bayesian analysis.*

The initial elaborations using Volcano analysis, using a fold-change cut off value of 3 and a *p* = 0.05, gave limited results. Only three enzymes passed the thresholds after 15 days of stress, namely ATP synthase subunit a, 30S ribosomal protein S4, and glutamine synthetase(GS; Benjamini–Hochberg corrected *p* = 2.1 × 10^-3^, 2.1 × 10^-3^, and 1.3 × 10^-3^, respectively), probably as a consequence of the high variability related to shotgun data-dependent analysis. Therefore, the proteomic results were discussed considering a fold-change threshold of 3 (on log 2 normalized abundances). This subset of proteins was loaded into the Pathway Architect add-in of Mass Profiler Professional. The list of proteins showing a fold-change of at least 3 in zinc-stressed plants only is reported in **Table 1**. Proteins are listed according to their accumulation trend and as a function of the time point considered. The Venn analysis on those proteins passing the fold-change cut off was next carried out, in order to dissect the changes specifically related to zinc toxicity from the variation induced by osmotic stress, being the former in common with soil salinity.

**FIGURE 3:**
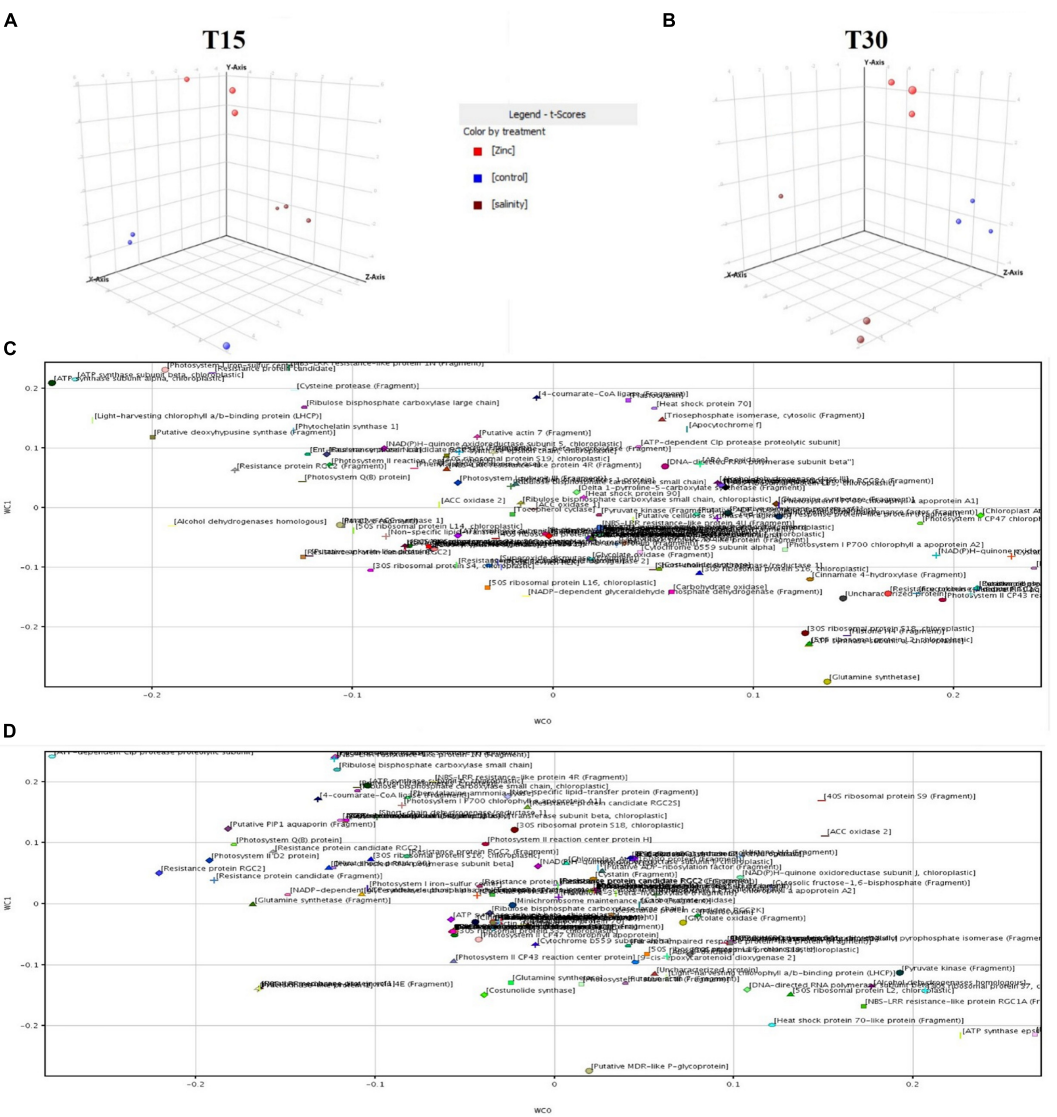
**Partial Least Squares Discriminant Analysis (PLS-DA) identification of proteins being significantly altered under zinc stress. (A,B)** are the hyperspace plots from PLS-DA models at 15 and 30 days, respectively, while **(C,D)** are the class prediction loadings on first (WC0, *x*-axis) and second (WC1, *y*-axis) hyperspace components.

To strengthen the outcome from fold-change and Venn analysis, the dataset was subjected to multivariate Partial Least Square Discriminant Analysis (PLS-DA) in Mass Profiler Professional. The loadings used to build the class prediction model were plotted according to their weight within the latent vectors, and the most relevant ones in predicting zinc-treated samples (i.e., those having a score of above +0.1 rather than below -0.1) were exported and recorded. In **Figure 3**, the distribution of each replication within the hyperspace of PLS-DA is given together with the plot of the class prediction loadings used to model covariance structures.

Finally, statistical confirmation regarding the involvement of the proteins identified in fold-change has been achieved using the “find minimal entities” naïve Bayesian analysis in Mass Profiler Professional. According to this approach, a target number of 30 proteins able to better explain differences between zinc treated samples from the others (forward selection algorithm; evaluation metric: overall accuracy = 100) was identified.

Interestingly, both covariance-based PLS-DA and Bayesian analysis outcomes included proteins already pointed out in the former fold-change analysis. Information regarding those proteins evidenced by multivariate statistics is given in **Table 1** together with results from fold-change analysis.

The results, grouped according to their ontology and biological meaning, are presented as follows more detailed.

### Photosynthesis and Electron Transport Chain

The Far-red impaired response protein-like protein, PSI chlorophyll *a* apoproteins A1 and A2 (PSI-A1 and PSI-A2)several NAD(P)H-quinone oxidoreductase subunits involved in the electron transport chain in chloroplast, exhibited changes in abundance as a consequence of zinc stress. Additionally, the photosystem I assembly protein YCF3 is required to assembly of PSI by interacting with its subunits (Naver et al., 2001). Also the biosynthesis of plastoquinone (PQ) might be altered under zinc exposure, although with contrasting results. The 37 kDa inner envelope membrane protein, a pivotal component in chloroplast development, and PQ biosynthesis (Motohashi et al., 2003), was down-accumulated at 15 days of treatment; conversely, the MPBQ/MSBQ transferase that methylates 2-methyl-6-phytylbenzoquinol for the production of tocopherols or PQ, was up-accumulated after 30 days of stress.

### Energy Metabolism

Besides these chloroplastic responses, the glycolytic pathway was also affected by the treatments considered. The increase in cytosolic triose phosphate isomerase (TPI) was consistent with the results from Riccardi et al. (1998) who related TPI accumulation to drought stress and water availability. An increase of TPI provides evidence in the formation of glyceraldehyde-3-phosphate, which can positively affect the energy level in plant and thus supports the plant growth (Sharma et al., 2012a). The up-accumulation of the downstream following glycolytic enzymes NAD(P)H-dependent glyceraldehyde-3-phosphate dehydrogenase (GAPDH) and pyruvate kinase, common to salinity and zinc, confirms the increase in catabolic energy production.

### Transcription and Protein Metabolism

The clearest evidence regarding the involvement of nitrogen compounds in response to zinc stress was related to GS. This protein was up-accumulated under both salinity and zinc stress, suggesting an improved assimilation of ammonium.

Additionally, several ribosomal proteins changed their abundance under zinc stress, and two heat shock proteins related to protein folding (HSP70, HSP90) were up-accumulated in response to zinc; these molecular chaperones assist the correct folding and contrast protein degradation in various abiotic stresses. The HSP90 then decreases after 30 days of zinc stress, probably as a consequence of plant adaption.

The protein metabolism was affected by zinc at different levels. MatK, a protein involved in in post-transcriptional processes during chloroplast development, was down-accumulated at 15 days. A zinc finger-homeobox (ZF-HD) protein-like protein, acting as transcription factor in the DNA binding, was not detected at the first time point. Indeed, zinc plays a role in plastidial transcription as a cofactor for RNA polymerase and ZFs-containing nucleic acid-binding proteins, as well as the up-accumulated DNA-directed RNA polymerase.

In addition, the reduced level of plastidial fructokinase-like proteins, the target of plastidial thioredoxin TRX z, regulated the plastid-encoded RNA polymerase dependent transcription in plastids (Arsova et al., 2010).

The down-accumulation of putative ankyrin-like protein (AKR), related to transcriptional regulation and signal transduction, is known to play an important role in protein–protein interaction in biotic and abiotic responses (Voronin and Kiseleva, 2008). In *Arabidopsis*, a reduced expression of AKR has been associated to the regulation of antioxidant metabolism that is shared by both disease resistance and stress responses (Yan et al., 2002).

### Phytohormones

Besides signaling, the key enzymes for the biosynthesis of ethylene were among our differential results. ACS catalyzes the formation of ACC, and then converted in ethylene by ACO. Two different isoforms of ACS have been pointed out in this work: ACS1 was down-represented after 15 days of stress, while ACS2 was up-represented during all the experiment. ACS isoforms have different activities in response to different stimuli: ACS1 may function as regulator of other ACS enzymes (Wang et al., 2002). ACO1, however, was up-accumulated in both salinity and zinc stress. Finally, a putative ethylene receptor (ETR1) was also up-accumulated at the first sampling event in lettuce exposed to zinc. The enhanced biosynthesis of ethylene during the whole experiment is not surprising, taking into account that this hormone has been linked to both ROS and heavy metals stress (Steffens, 2014).

Furthermore, both abscisic acid (ABA) biosynthesis and degradation were strongly affected by zinc treatment. ABA is synthetized in the cytosol from xanthoxal and is related to developmental processes, responses to abiotic stress (drought, cold, salt, and wounding), seed development, and dormancy. The carotenoid cleavage dioxygenases (CCDs) catalyze the selective oxidative cleavage of carotenoids, leading to apocarotenoids and then to ABA. CCDs can exert their role by modulating the phytohormones network (Cazzonelli, 2011). In our experiments, CCD2 presented a reduced level of expression starting from 15 days of treatment, while CCD1 decreased at 30 days. The nine-*cis*-epoxycarotenoid dioxygenase (NCED), a subfamily of CCD for ABA biosynthesis exhibiting different specificity for the double bound that it cleaves (Lu and Li, 2008), presented a trend consistent with CCDs. NCED3 (related to environmental stress response) and NCED2 were down accumulated, while NCED4 was up-accumulated; however, this latter seems to be involved in thermal inhibition and no evidences are reported regarding water stress (Huo et al., 2013). The P450 monooxygenase enzyme ABA 8-oxidase (ABA8ox), involved in the oxidative degradation of ABA, was found up-accumulated.

The transcription factor MYC2 is a basic helix-loop-helix type protein that modulates different aspects of jasmonic acid signal transduction by regulating the transcription of its target genes (Zhai et al., 2013), is involved in mediating the cross-talk between jasmonate, ethylene, gibberellin, and light signaling, and it is related to ABA response (Finkelstein, 2013). According to our results, MYC2 decreased during zinc stress consistently to ABA biosynthesis, which is negatively regulated.

In association to ABA, the gibberellins metabolism was engaged in this study consistently with DELLA proteins accumulation. Gibberellins are known to regulate, among others, the response to abiotic stress. The down-accumulated germacrene A (GA) insensitive Dwarf1 A (GID1) protein is a gibberellin nuclear receptor that can interact with DELLA proteins to bind the active GA form. In agreement with the accumulation of DELLA proteins, ent-kaurene oxidase 1 (KO), catalyzing the second step for the plastidial synthesis of GA (Swain et al., 2005), was down-accumulated.

### Membrane Transport and Cell Wall Metabolism

Two enzymes belonging to lignin biosynthesis were differentially measured after zinc exposure: pectin acetylesterase (PAE) was up-accumulated while a putative cellulose synthase (CesA) was down-accumulated.

Besides cell wall, transport across the membrane was also affected by zinc treatment. Plants can cope with osmotic stress by means of the water channels aquaporins; the putative PIP2 aquaporin was identified in reduced abundance, while PIP1 aquaporin was up-accumulated under both zinc and salinity stress. The ABC cassette transporters YCF1 and MDR-like *P*-glycoprotein were also up-accumulated.

### Phenylpropanoids Biosynthesis

The phenylpropanoids pathway was altered as a response to zinc stress, although with differences between phenolic classes. Surprisingly PAL, the upstream enzyme in phenylpropanoids biosynthesis, was measured at reduced amounts in plants although flavonoid accumulation is induced by saline stresses (Colla et al., 2013). The same trend was observed for 4-coumarate-CoA ligase (4CL), the key enzyme in chalcone synthesis, under both salinity and zinc stress. Conversely, the flavanone-3-beta-hydroxylase (F3H), another enzyme of the phenylpropanoid pathway that catalyzes the formation of flavonols, was found up-accumulated at both time points. Finally, the increased amount of cinnamate-4-hydroxylase (C4H) suggests the biosynthesis of hydroxycinnamic acids.

### Other Responses to Osmotic/Oxidative Stress

Several enzymes related to secondary compounds synthesis changed their abundance in zinc-stressed plants, like for tocopherol cyclase (TC) that exhibited low accumulation.

Regarding sesquiterpene lactone biosynthesis, two key enzymes were up-accumulated after exposure to zinc. The increase in GA synthase (GAS) LTC1, an enzyme involved in the biosynthesis of secondary metabolites such as phytoalexins, was observed. The second sesquiterpene lactone biosynthetic enzyme that was found up-accumulated is costunolide synthase (CYP71BL2), supposed to act as a phytoalexin and to modulate oxidative stress. Consistently, the degrading enzyme GA oxidase (GAO), catalyzing the formation of an acid group in GA, was decreased under salinity and zinc stress.

The accumulation of proline can be expected considering the up-accumulation of delta 1-pyrroline-5-carboxylate synthetase (P5CS).

Among plant antioxidants, L-ascorbate has a key role in the mitigation of ROS. The L-galactono-1,4-lactone dehydrogenase (L-GalLDH) increase, converting L-GalL into ascorbate.

The terpenoids biosynthetic pathway was also affected by the stress considered. The enzyme isopentenyl pyrophosphate:dimethyllallyl pyrophosphate isomerase (IDI), a protein that catalyzes the isomerization of isopentenyl pyrophosphate and dimethylallyl pyrophosphate in terpenoid biosynthesis, was increased during the experiments.

Finally, some resistance proteins like the resistance gene candidate (RGC) and the non-specific lipid transfer protein (nsLTP) were also imbalanced in response to zinc exposure.

## Discussion

### Zinc-Related Effects

The comparison of results from zinc-treated and salt-treated plant samples let to identify those changes at proteome level, specifically induced by zinc. The up-accumulation of Far-red impaired response protein-like protein (a positive regulator of the genes for porphobilinogen synthase) and PSI-A1 and PSI-A2 indicate an increase in chlorophyll biosynthesis. Therefore, the plant photosynthetic process was strongly affected, mainly at chlorophyll biosynthesis and at photosystem I level. Coherently, the reduction in plastocyanin and photosystem II reaction center protein H, suggested a modulation of photosynthesis toward PSI. Since PSI is the terminal electron carrier in the chloroplast, coupled to ROS-scavenging and/or photophosphorylation in the chloroplast (Sharma et al., 2012b), these findings can be correlated to the osmotic stress. A confirmation of this hypothesis was found in the concomitant up-accumulation of ATP synthase subunits.

The glycolytic process was stimulated by the treatment with zinc, probably as a supply of energy substrates. Analogously, ammonium assimilation (through stimulation of GS) was induced by zinc in soil. This latter step provides with an essential nitrogen precursor for almost all nitrogenous compounds; indeed, the key role of GS in contrasting saline stress has been pointed out by Teixeira and Fidalgo (2009). Coherently, transcription and protein synthesis, as well as proteolysis, were strongly affected by the treatment with zinc. Proteolysis is a part of the regulatory mechanism for a broad spectrum of cellular processes affecting not only the stability of key metabolic enzymes but also on the removal of terminally damaged polypeptides. Several of these processes are connected to ATP-dependent proteases. In plants, the balance of cystatin/cysteine protease plays an essential role in protein turnover and in the response to biotic/abiotic stresses (Benchabane et al., 2010). In our experiments, both cystatin and cysteine protease were up-accumulated after zinc exposure.

As expected, the hormone network seems to be altered by the treatments. Several proteins involved in plant hormone metabolism and cross-talking were identified in our experiments, including ABA, gibberellin, and ethylene metabolism. According to our results, the crosstalk of plant hormone signaling should have a pivotal role in stress hindering and osmotic adaptation. Besides the specific stimulation of ethylene biosynthesis enzymes, ABA biosynthesis was induced after 15 days of stress, via the up-accumulation of some CCD and NCED proteins. At the same time point GA synthesis did not show changes. Then, ABA synthesis seemed to decrease markedly, while GA followed the same trend but with a less evident decrease. The coordinated change in ABA/GA ratio we expect from biosynthetic enzymes was consistent with the assumption regarding their antagonistic role in plant response to stresses. GIGANTEA (GI), a protein related to GA signaling and involved in oxidative stress tolerance, cold stress responses, and carbohydrate metabolism (Molas et al., 2006), was down accumulated in response to zinc. These findings are consistent with previous literature reporting decreased amounts of GI under salt treatment (Park et al., 2013). The kinase receptor cysteine-rich RLK, involved in hormonal signaling in several species, was up-accumulated, suggesting that signal transduction is also involved.

Membrane trafficking was affected at both aquaporins and ABC cassette transporters level. The accumulation of PIP1 aquaporin only, a transporter actually reported as the more efficient water channel (Kaldenhoff and Fischer, 2006), was coherent with the treatment applied. ABC transporters play an important role in growth, plant nutrition, and development, response to abiotic stress and stimuli. It has been reported that they might have a possible role in metal accumulation in the vacuole, as detoxification mechanism (Luque-Garcia et al., 2011).

Although phytochelatins are described to play an important role in heavy metal detoxification in plants, phytochelatin synthase 1 (PCS1) was down-accumulated under both salinity and zinc stress. Interestingly, some authors (Lee et al., 2003) reported that the overexpression of PCS led to hypersensitivity toward cadmium and zinc stress in *Arabidopsis*.

Several other enzymes acting in secondary metabolism at different levels have been regulated in lettuce as a response to zinc exposure. TC is involved in tocopherol synthesis at the inner chloroplast envelope level (Cheng et al., 2003), representing a plant response to deal with the accumulation of ROS then protecting from lipid peroxidation. Surprisingly, this enzyme was down-accumulated throughout the whole experiment, in agreement to the low abundance of the 37 kDa inner envelope membrane protein.

Other enzymes involved in secondary metabolism included sesquiterpene lactones, proline, ascorbate and terpenoids biosynthesis, and therefore are consistent with zinc stress. The phenylpropanoids pathway likely switches from flavonoids to flavonols and hydroxycinnamic acids synthesis, thus accumulating these latter. Sesquiterpene lactone phytoalexins were also induced in lettuce leaf, as well as resistance proteins and proline biosynthesis. Considering that LTC1 converts farnesyl diphosphate to GA, a Mg^2+^ ion binding compound, its role in zinc stress might be related to GA metal binding capacity and/or to the phytoalexin activity of sesquiterpene lactones.

The accumulation of proline has the role of protecting proteins and quenching ROS, by acting as organic osmolyte and contributing to osmotic adjustment (Borgognone et al., 2013). The role of ascorbate in stress mitigation can be also related to ROS scavenging, being oxidative stress a consequence of osmotic stress.

Although the role of terpenoids in contrasting osmotic stresses has been already reported (Lucini et al., 2015), IDI is a divalent metal ion-requiring enzyme and seems to bind Mg^2+^ and Zn^2+^. Therefore, its involvement in the response to zinc could be actually related to the stress induced.

Summarizing, the coordinate response to zinc stress included more expected responses, like the imbalance of hormone network rather than the need to cope with osmotic and oxidative stress, as well as some more specific and more surprising changes like the modulation of the photosynthetic activity and the involvement of proteolysis.

### Changes During Time

The proteomic response of lettuce throughout our experiment, suggests that the plant actually exhibits an adaption to stress over time. Overall, 58 proteins (34 of which could be ascribed to zinc only) were up-accumulated after 15 days of stress, to decrease after 30 days (27 proteins up-regulated, 12 of which related to zinc). Regarding the down-regulated ones, 43 proteins were identified at 15 days (23 those related to zinc only) while 62 showed the same trend (25 those from zinc stressed plants only) after 30 days.

Some key processes, like those catalyzed by GS and ATP synthase (both up-accumulated) and PCS (down-accumulated), did not show variation in trend during the experiment. However, the most of the proteins changed their abundance during the time course.

#### Early Response to Stress

The involvement of PSI electron transport chain was evident after 15 days of stress, where chlorophyll *a* apoproteins and far-red impaired response protein-like protein were up-accumulated, to decrease then at 30 days. At this latter time point, these proteins abundance in stressed plants was even below the level of control plants. Consistently, plastocyanin followed an opposite trend than chlorophyll apoproteins and far-red impaired response protein-like protein.

Sesquiterpene lactones synthesis was induced at 15 days, as proven by the increase in both GAS (concurrent to a down accumulation of GAO and costunolide synthase, to return at the same level as in the control, at 30 days. Similar trend was observed for resistance proteins, CesA and heat-shock proteins (excepting NBS-LLRs that were down accumulated at both time points and HSP90 that decreased below the reference control at 30 days). Analogously, the alcohol dehydrogenase (ADH) mediated coping of reactive nitrogen species, was observed at the earlier time point. The energy supply via glycolysis was stimulated at 15 days of stress, being TPI and GAPDH up-accumulated; the former was then down accumulated after 30 days.

#### Late Response to Stress

The accumulation of some enzymes, known to be implicated in plant coping with stress, was induced after 30 days of stress. The biosynthesis of ascorbate via L-GalLDH and the synthesis of MDR-like ABC cassette are both induced at the later sampling point.

Phenylpropanoids biosynthetic pathway was down-accumulated at both time points, being both 4CL and PAL reduced, although an increase in hydroxycinnamic acids can be forecast at 30 days, where C4H level increased.

### Comparison of Zinc-Related versus Salinity-Related Changes

Although the proteins involved in the response to zinc stress and to salinity were substantially the same, their abundance changed between the two treatments. As a general consideration, lettuce response to zinc was more prominent at the first sampling point, yet showing a faster adaptation than under NaCl stress after 30 days.

Regarding hormone profile, it can be pointed out that the down accumulation of GID1 A protein occurred already after 15 days of stress in zinc, while was later in salt stressed plants. Consistently, DELLA 2, a protein being accumulated under low GA levels, was up-accumulated in zinc-stressed plants only. These evidences suggest that zinc stress has a more prominent effect on GA metabolism than salinity. In addition, ethylene biosynthetic enzymes were more abundant under zinc conditions.

The increased abundance of PIP2 aquaporin and MDR-like glycoprotein ABC cassette in zinc-stressed plants after 15 days, followed by reduced amounts after 30 days, suggest that lettuce better managed to cope with ion-related electrolyte-water imbalance under zinc stress than under saline conditions. Similarly, the induction of sesquiterpene lactones, proline, and ascorbate was more pronounced in zinc stressed plants, being biosynthetic enzymes up-accumulated after 15 days; in all cases, except for the latter one, protein abundances decreased to basal level after 30 days.

The enhanced engagement of PSI was more marked in zinc-treated plants after 15 days, as well as ADH, glycolytic enzymes and ATP synthase; again, the plant showed an adaption to stress and in most of the cases enzymes decreased to be less abundant under zinc than NaCl conditions.

Finally, in agreement with the general trend above reported, heat shock proteins were more abundant in zinc-stressed lettuce at 15 days, to decrease at 30 days, when GS became down accumulated.

## Conclusion

The effect of zinc stress in lettuce, in comparison with salt stress, was investigated at proteome level via shotgun data-dependent mass spectrometry. The analysis of proteins exhibiting a fold change of 3 as minimum (on log 2 normalized abundances), revealed the involvement of photosynthesis, via stimulation of chlorophyll synthesis and enhanced role of photosystem I, as well as stimulation of photophosphorylation. Glycolytic supply of energy substrates, together with ammonium assimilation (through formation of GS) were also stimulated by zinc in soil. Similarly, protein metabolism (at both transcriptional and ribosomal level), heat shock proteins, and proteolysis were affected.

The treatment and the time point considered affected plant hormone profile: ethylene biosynthesis was enhanced throughout the whole experiment, while production of ABA was induced at the earlier time point to decrease markedly, and gibberellins were decreased at the later time point.

Besides aquaporin PIP2 synthesis, other osmotic/oxidative stress related compounds were enhanced under zinc stress, i.e., proline, hydroxycinnamic acids, ascorbate, sesquiterpene lactones, and terpenoids biosynthesis.

Most of the proteins pointed out were in common between zinc and NaCl stress, although the Zn-related response was quantitatively different and anticipated in time. Finally, lettuce plants showed an adaptation after 30 days of stress that was more evident in the case of zinc.

## Conflict of Interest Statement

The authors declare that the research was conducted in the absence of any commercial or financial relationships that could be construed as a potential conflict of interest.

## Supplementary Material

The Supplementary Material for this article can be found online at: 
